# A Rare Case of Small Bowel Obstruction in Pregnancy Due to Adenocarcinoma

**DOI:** 10.7759/cureus.53124

**Published:** 2024-01-28

**Authors:** Zainab Almulhim, Sharifah Othman, Mosab Alarfaj, Nasreen Hamadah, Omar Bamalan, Faris Alanazi

**Affiliations:** 1 Department of Obstetrics and Gynecology, Imam Abdulrahman Bin Faisal University, Dammam, SAU; 2 Department of Obstetrics and Gynecology, King Fahad University Hospital, Al Khobar, SAU; 3 Department of Surgery, Imam Abdulrahman Bin Faisal University, Dammam, SAU; 4 Department of Surgery, King Fahad University Hospital, Al Khobar, SAU; 5 College of Medicine, Imam Abdulrahman Bin Faisal University, Dammam, SAU

**Keywords:** small-bowel obstruction, hyperemesis gravidarum, pregnancy, small bowel adenocarcinoma, small bowel carcinoma

## Abstract

Small bowel obstruction (SBO) rarely occurs in pregnancy, primarily due to the adhesions resulting from previous abdominal surgery. However, malignancy causing SBO during pregnancy is exceedingly rare. We present a case of a 34-year-old pregnant woman who was recently diagnosed with small bowel disease at 19 weeks and two days of gestation and initially managed conservatively. Diagnostic procedures, such as endoscopy or colonoscopy and enterography magnetic resonance imaging (MRI), were postponed due to her pregnancy. With recurrent episodes of worsening symptoms, the patient underwent multiple admissions, during which an abdominal X-ray was performed, revealing dilated loops of the small and large bowel, highly suggestive of SBO. Subsequently, a plain abdominal MRI revealed a stricture in the left lower quadrant, resulting in SBO. Given the absence of a fetal pulse, the patient underwent an emergency laparotomy. Surgical resection involving part of the mass in the terminal ileum was performed, followed by a primary side-to-side anastomosis. Histopathological examination of the resected tissue confirmed the presence of small bowel adenocarcinoma. The successful surgical resection and subsequent histopathological confirmation emphasized the importance of prompt diagnosis and appropriate management. This case underscores the challenges faced in diagnosing and managing small bowel obstruction during pregnancy, particularly when malignancy is the underlying cause. It highlights the need to balance diagnostic investigations with fetal safety. Multidisciplinary collaboration between obstetricians, surgeons, and radiologists is crucial in navigating the complexities of managing such cases and ensuring optimal outcomes for both the mother and the fetus.

## Introduction

Small bowel obstruction (SBO) often results from adhesions due to prior abdominal surgery. Rarely, it is also caused by malignancy, hernias, volvulus, or intussusception. Conservative management is the preferred approach for SBO caused by adhesions; however, immediate surgery is required for SBO resulting from other causes (e.g., complicated peptic ulcer disease with complete obstruction) [1]. Moreover, SBO is a rare clinical problem experienced during pregnancy, with an occurrence rate of 0.001-0.003%, with adhesions accounting for 70% of cases [2]. In the case of SBO during pregnancy, the safest means to protect the fetus is through nonoperative methods while avoiding radiation and harmful medications, though surgical management might also be required [1,3]. Proper diagnosis and treatment of this condition in pregnant women are vital since it is associated with significant maternal and fetal mortality [4-6]. Here, we present a rare case of SBO in pregnant women due to malignancy.

## Case presentation

A 34-year-old Saudi female was referred to the emergency room (ER) of a university hospital in Dammam, Saudi Arabia, at 16 weeks and two days of gestational age with symptoms of left iliac fossa pain, watery diarrhea, nausea, and vomiting for the past three to four months. In addition, she had a history of loss of appetite and a weight reduction of about 10 kilograms, with no fever, headache, bleeding in the stool, urinary symptoms (e.g., frequency, dysuria), blurred vision, and change in the level of consciousness.

The first time she sought medical attention was two months before the admission. She visited a hospital in the United States with complaints of nausea, vomiting, severe abdominal pain, diarrhea, and weight loss (about 10 kilograms in two months) at 10 weeks of gestational age and was referred to gastroenterology. On ultrasound, normal fetal heart rate and movement were observed. On laboratory results, abnormal levels of leukocytosis and hypoalbuminemia were noted. Magnetic resonance imaging (MRI) of abdomen and pelvis without contrast revealed distal ileum and right colon concerning for inflammatory bowel disease (IBD), or an infectious disease (e.g., tuberculosis), demonstrating signs of active ileitis and proximal colitis.

Colonoscopy found a localized area of minimal erythematous mucosa in the rectum, and biopsies were taken for histopathological examination. The results of histopathology showed minimal focal acute colitis, which was negative for granulomas and dysplasia, and benign colonic mucosa, which was negative for active inflammation, granulomas, and dysplasia. She was diagnosed with undetermined IBD, although initially suspected to be Crohn's disease. She was started on prednisolone daily, but the dosage was tapered down due to her pregnancy. She had a transient improvement of symptoms following steroid medications.

From the university’s hospital ER, she was referred to gastroenterology for consultation, as all her basic investigations were found to be normal and her fetal ultrasound scan report showed no abnormality, with no complaints reported related to her pregnancy, such as vaginal bleeding or labor-like pain. The abdominal ultrasound was requested and revealed a mild small bowel dilatation, thick intestinal wall, and mild ascites. Further, additional radiological investigations were needed to rule out small bowel disease. However, due to her pregnancy and to avoid radiation risk to the fetus, she was suspected to have colitis based on her past medical report, which could be a result of either an infection or inflammatory bowel disease (IBD). During hospitalization, she was treated with pantoprazole, paracetamol, ringer lactate, and ceftriaxone for five days. On the fifth day from the date of admission, she was discharged in good and stable condition.

Three days later, she was admitted again to the ER with complaints of constipation, nausea, and vomiting for three days with reduced appetite. There were no urinary symptoms, abdominal pain, watery diarrhea, and rectal discharge or bleeding. Hypokalemia was noted in her basic investigations. Hence, a joint meeting with the GI team resulted in a recommendation against requesting diagnostic endoscopy or colonoscopy and recommended symptomatic relievers till delivery with enterography, and MRI after delivery. She was treated with domperidone, paracetamol, Normacol, and pantoprazole. Following treatment, she experienced relief from constipation, and her electrolytes were balanced. On day five of hospitalization, she improved and was discharged with home medications such as pantoprazole, metoclopramide, and paracetamol.

Nevertheless, on the next day, she was again admitted to the ER at 18 weeks of gestational age with the symptoms of nausea, greenish vomiting, obstipation, generalized abdominal pain with distention, bloating, generalized weakness, signs of dehydration, and loss of appetite. She had no history of fever, headache, change of consciousness, syncope, shortness of breath, palpitation, chest pain, jaundice, urinary symptoms, melena, rectal discharges, or rectal bleeding. The patient was not able to tolerate oral intake and was fatigued. Also, her pregnancy was uncomplicated and fetal movement was normal. There was no history of vaginal bleeding, pelvic pain, labor-like pain, or leaking. She was under her regular medications, prednisolone, navidoxine, metoclopramide, and pantoprazole.

On investigation, ketone and potassium levels were observed as +4 and 2.7, respectively. So, she was treated with potassium chloride 40 mEq in normal saline. The GI team sought surgical consultation as they suspected the case to be small bowel obstruction (SBO) rather than steroid withdrawal.

Furthermore, no improvement in passing stool was observed following the administration of regular glycerin suppositories and enemas. The team inserted a nasogastric tube and administered it with fluids, and IV analgesics were given to retain the normal size of the small bowel. 

On examination, vital signs were within normal reference and the patient appeared cachectic. There was no respiratory distress or jaundice. The abdomen was soft, lax, and distended, but not tender. In addition, resonant with percussion and bowel sounds were felt. No rebound tenderness and guarding were observed. The chest was clear, with no added sound. Moreover, the rectal examination revealed no stool with stimulation, bleeding, and fistula. There was no vaginal bleeding or leaking.

On laboratory investigations, only renal function test (RFT) and complete blood count (CBC) were remarkable, as shown in Table 1.

**Table 1:** Demonstrating some of the ordered investigations for the patient. *Significant abnormalities.

Investigations	Results	Reference ranges
Hemoglobin level	11.6 g/dL*	12.0-16.0
White blood count	6.8 k/ul	4.0-11.0
Platelet count	280 k/ul	140-450
Potassium level	2.5 mEq/L*	3.5-5.1
Sodium level	135 mEq/L*	136-145
Chloride level	105 mEq/L	98-107
Creatinine level	0.17 mg/dL*	0.6-1.3
Blood urea nitrogen	9 mg/dL	7.0-18.7
Calcium level	8 mg/dL*	8.5-10.1

**Figure 1:**
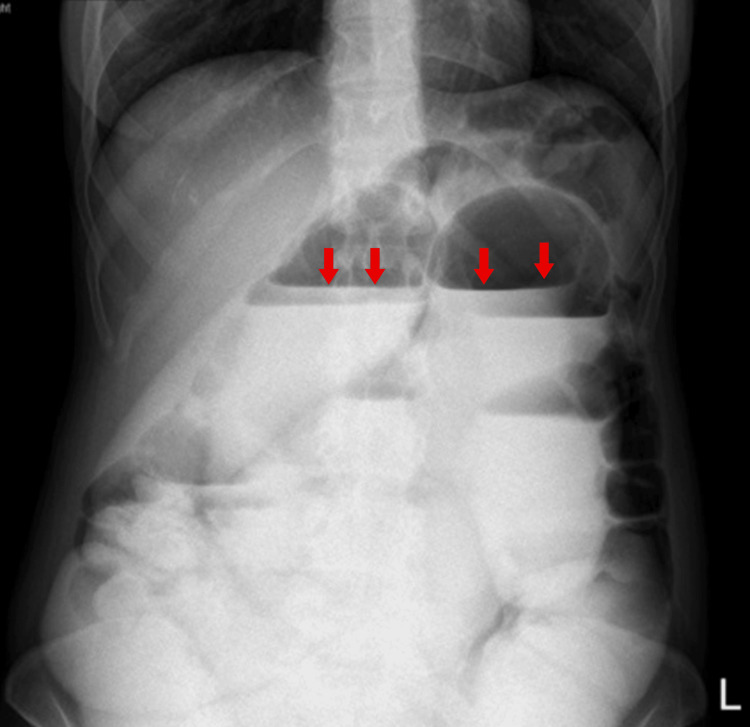
Abdominal X-ray demonstrated results indicating the presence of small bowel obstruction (i.e., air-fluid levels and dilated bowel) (marked by a red arrow).

**Figure 2:**
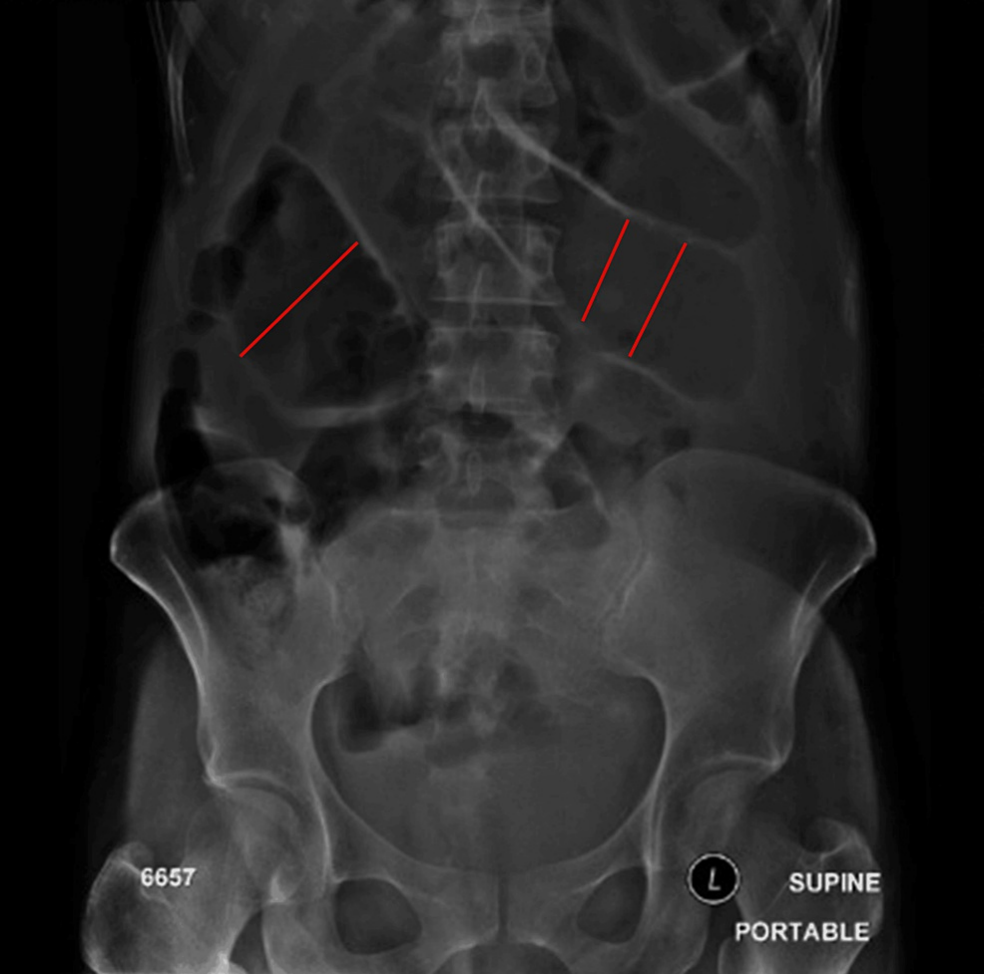
Abdominal X-ray demonstrated abnormal results indicating the presence of small bowel obstruction (i.e., dilated bowel) (marked by red lines).

Other findings, such as amylase, lipase, urinary analysis, and urinary culture, were within normal reference. Furthermore, pelvic ultrasound showed the presence of a fetal heart, normal measurements according to gestational age, adequate liquor, and normal placental texture. As repeated abdominal ultrasounds were suspicious, an abdominal X-ray and MRI were ordered. The supine abdominal X-ray revealed abnormal findings, such as dilated loops of the small and large bowel, and it was highly suggestive of SBO (Figures 1, 2).

**Figure 3:**
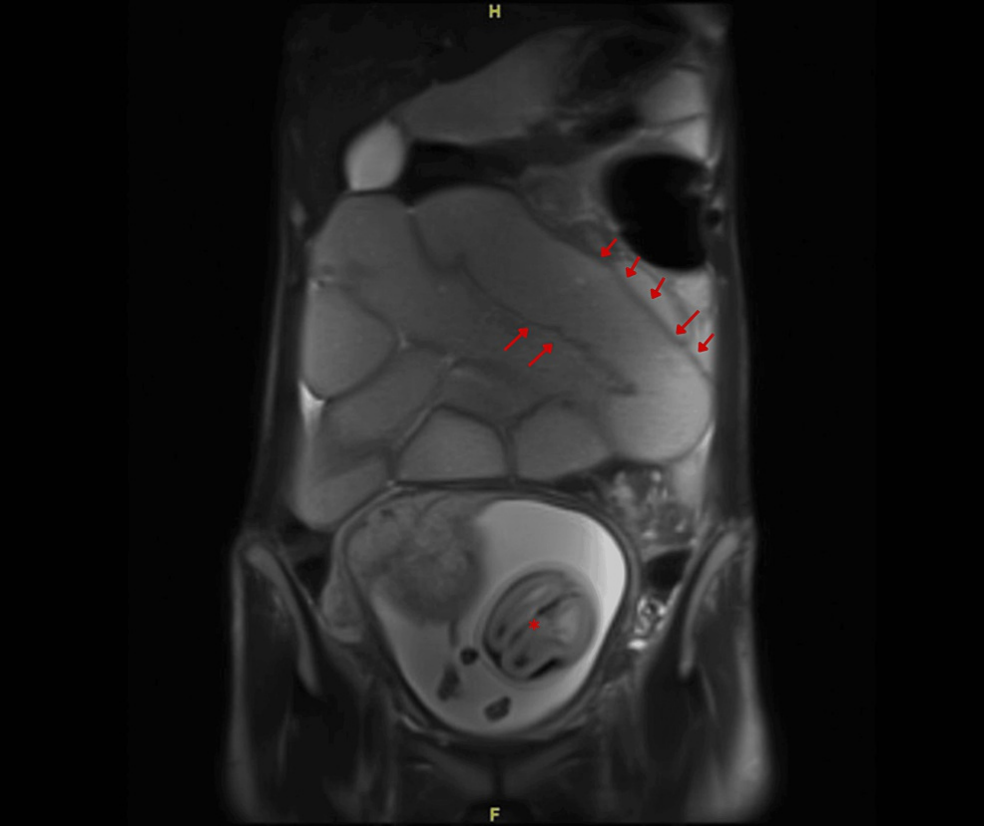
MRI of the abdomen without IV contrast showed a dilated small bowel reaching 6.4 cm (highlighted by the red arrows) and a fetus (marked by an asterisk).

On the sixth day, a plain abdominal MRI revealed that the stomach and duodenum collapsed, and small bowel dilation with a transition point was seen at the left lower quadrant. At the transition point, there is a focal area of circumferential wall thickening with luminal narrowing. There was no frank evidence of free fluid. The MRI report stated that a stricture was noted at the left lower quadrant and resultant SBO (Figure 3).

Based on these findings, she was diagnosed with complete small intestine obstruction. On the seventh day, she was 19 weeks and two days of gestational age, by which time her fetal pulse was absent and she was diagnosed to have missed abortion. She was evaluated clinically and using water-soluble solutions (gastrografin) with serial ultrasounds, alongside medical management. Unfortunately, no improvement was observed in her symptoms despite these measures. As a result, the colorectal surgical team decided to proceed with an emergency laparotomy and surgical resection of the mass located in the terminal ileum with primary side-to-side anastomosis. The surgery was performed on the 10th day from the date of the last admission. On opening the surgical site, ileum mass was observed at 40-45 cm from the ileocecal junction. There was proximal bowel distension with distal bowel collapse from the mass and multiple mesenteric lymph nodes. Free turbid fluid in the peritoneum was noted.

**Figure 4:**
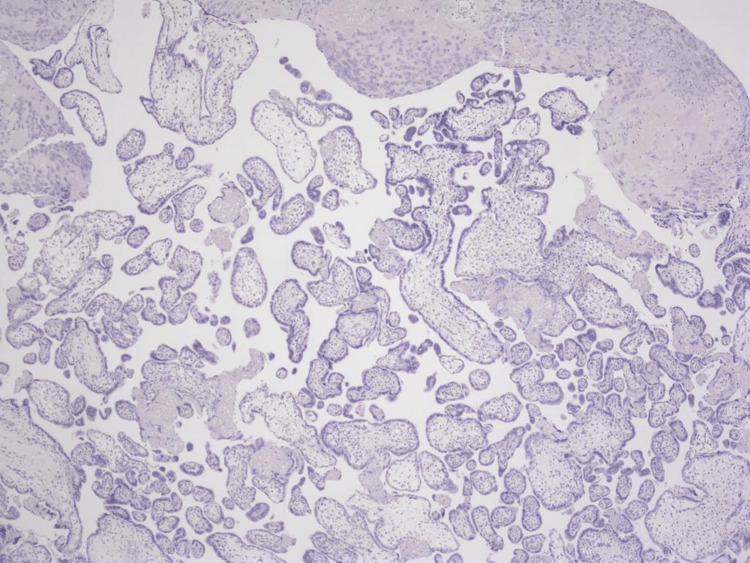
Histopathology slide from the mass resected at the terminal illum showing moderately differentiated adenocarcinoma.

The patient was shifted to the intensive care unit following the surgery. Histopathological examination showed a moderately differentiated adenocarcinoma with negative lymph node metastasis and negative cytology for malignancy in ascites fluid (Figure 4).

On the fifth postoperative day, she was administered misoprostol 400 mg vaginally every three hours for four doses. The patient aborted entirely with no complications. On the 13th postoperative day, she was discharged in good condition. She was transferred to a higher center for positron emission tomography (PET) scan and chemotherapy. The patient was referred to another specialist hospital and is currently undergoing adjuvant chemotherapy with FOLFOX (i.e., a regimen that is a combination of folinic acid, fluorouracil, and oxaliplatin).

## Discussion

Diagnosing small bowel obstruction (SBO) during pregnancy poses a significant challenge due to the resemblance of symptoms and physiological changes associated with pregnancy. The enlargement of the uterus and hormonal shifts can alter SBO symptoms, leading to confusion with other differential diagnoses. Furthermore, concerns about fetal radiation exposure restrict the use of radiological imaging in pregnancy, thereby complicating the diagnosis of SBO and potentially causing delays in management. Typical symptoms of SBO include nausea, vomiting, abdominal pain, abdominal distention, and constipation [4,7,8]. In our case, the patient initially presented with similar symptoms, along with constipation and weight loss. It is important to note that these symptoms can also be associated with other conditions, such as acute pancreatitis, inflammatory bowel disease (IBD), acute cholecystitis, and hyperemesis gravidarum.

While abdominal CT with contrast is usually the preferred imaging modality to confirm SBO suspicion, MRI has been recognized as a safe and valuable alternative for pregnant patients. In complex cases, MRI can be safely performed during the second and third trimesters, avoiding concerns about radiation exposure during early pregnancy. If MRI is unavailable, a supine abdomen X-ray can be considered a simple and low-risk option with minimal radiation exposure (less than 5 rads) [4,8,9]. In our case, an MRI without contrast was conducted during the patient's second trimester, confirming a complete SBO.

Conservative management is the initial approach for most patients, involving measures such as bowel rest, intravenous hydration, and nasogastric decompression, closely monitored by healthcare professionals. In the majority of cases, this conservative therapy leads to improvement. However, if conservative treatment proves ineffective or signs of bowel strangulation or fetal distress emerge, prompt surgical intervention becomes necessary, as it was in our case [8].

SBO commonly arises due to postoperative adhesions following previous abdominal surgeries [1,10]. However, our case did not have a history of abdominal surgeries. SBO caused by malignancy is an exceptionally rare occurrence, especially during pregnancy. Suspicion of SBO secondary to pregnancy should be considered if there are symptoms such as weight loss, night sweats, and fatigue, as observed in our case. Although these symptoms are not specific, additional workup and imaging should be considered [2]. The cause of SBO in our case was revealed to be small bowel adenocarcinoma (SBA) through histopathological examination. SBA is a rare type of tumor, and its incidence is increasing. It accounts for approximately 40% of all small bowel cancers, with the duodenum being the most commonly affected segment [11]. Previous studies have reported incidence rates of SBA in the duodenum, jejunum, and ileum, ranging from 55 to 82%, 11 to 25%, and 7 to 17%, respectively [12-14]. Although SBA primarily occurs in the duodenum, our case exhibited an uncommon occurrence of adenocarcinoma in the ileum. Recent studies have indicated that malignancy can lead to SBO in rare cases, accounting for 20% of SBO incidences [1,15]. While intestinal obstruction during pregnancy is a rare condition, it can result in significant maternal mortality (6-20%) and fetal mortality (20-26%) [4]. A literature review reported a total of seven fetal deaths in SBO cases during pregnancy, with three attributed to adhesive SBO and one to volvulus SBO. Adhesive SBO had a high incidence of fetal deaths during the second trimester [1]. In our case, fetal loss occurred during the second trimester due to SBO caused by adenocarcinoma.

## Conclusions

Our case was initially investigated with an abdominal ultrasound and managed conservatively due to the patient's pregnancy. However, further radiological investigations, such as X-ray and MRI, were delayed to avoid fetal radiation exposure. In addition, conservative management was chosen to avoid surgery during pregnancy. Nevertheless, as the symptoms worsened, further radiological investigations and surgical resection were eventually pursued. The histopathological examination confirmed the cause of SBO during pregnancy to be SBA, a rare tumor that is uncommon in the ileum. In cases of SBO, early diagnosis and appropriate surgical intervention should be a team’s priority, particularly when symptoms are worsening, to reduce the risk of maternal and fetal mortality.
